# Comparative study of melasma in patients before and after treatment based on lipomics

**DOI:** 10.1186/s12944-024-02130-z

**Published:** 2024-05-11

**Authors:** Yuan Zhu, Jinhui Xu, Xiuzu Song, Wenzhong Xiang

**Affiliations:** Department of Dermatology, Hangzhou Third People’s Hospital, Westlake Ave 38, Hangzhou, China

**Keywords:** Melasma, Lipidomics, Melasma area and severity index

## Abstract

**Background:**

Skin barrier alterations play a crucial function in melasma development. Past researches have demonstrated variations in lipid content between the epidermis of melasma lesions and normal tissues, along with the varied expression of lipid-related genes in melasma. This study aimed to analyze the lipidome profiles of skin surface lipids (SSL) in patients with melasma before and after treatment to understand associated abnormalities.

**Methods:**

Melasma was treated with tranexamic acid orally and hydroquinone cream topically. Disease was assessed using the Melasma Area and Severity Index (MASI), and the impact to life was evaluated with Melasma Quality of Life (MELASQoL) score. Epidermal melanin particles were observed using reflection confocal microscopy (RCM), whereas epidermal pigment and blood vessel morphology were observed using dermoscopy, and SSL samples were collected. Specific information regarding alterations in lipid composition was obtained through multivariate analysis of the liquid chromatography-mass spectrometry data.

**Results:**

After treatment, patients with melasma exhibited decreased MASI and MELASQoL scores (*P* < 0.001); RCM revealed reduced melanin content in the lesions, and dermoscopy revealed fewer blood vessels. Fifteen lipid subclasses and 382 lipid molecules were identified using lipidomic assays. The expression levels of total lipids, phosphatidylcholine, and phosphatidylethanolamine in the melasma lesions decreased after treatment (*P* < 0.05).

**Conclusion:**

This study revealed alterations in the SSL composition after effective melasma treatment, suggesting a compensatory role for lipids in melasma barrier function. The mechanism involving SSL and the lipid barrier, which influences melasma’s occurrence, needs further elucidation.

**Supplementary Information:**

The online version contains supplementary material available at 10.1186/s12944-024-02130-z.

## Introduction

Melasma is a chronic, symmetrical facial pigmentation. The pathogenesis of melasma is complex and is currently believed to be related to hyperplasia of the local vasculature, chronic inflammation, impairment of the skin’s barrier function, and other proposed hypotheses [1]. Prior research has demonstrated that the melasma lesion area had much higher levels of total lipids (including phosphatidylic acid, ceramide (CER), and phosphatidylserine) than non-lesioned skin [2]. .

Lipids are characterized as hydrophobic or amphoteric small molecules, and they are grouped into eight distinct categories as identified by LIPID MAPS® [3]. As the primary constituents of an organism’s membrane framework, lipids play dual roles in both signaling and as a source of energy. Skin surface lipids (SSL) form an appropriate lipid barrier for the epidermis and play an essential role in maintaining moisture and elasticity [4]. Disturbances in the number and composition of local lipids have been shown to contribute significantly to skin barrier damage and are key factors in the development of various skin diseases [5].

Lipidomics employs a high-throughput analytical approach to comprehensively investigate changes in the composition and expression of lipid molecules within living organisms. It was first introduced in 2003 [6]. Through lipidomics, alterations in lipid composition have been suggested to contribute to the occurrence and progression of dermatosis like atopic dermatitis (AD) [7], psoriasis [8, 9], and acne [10–12].

Research on the changes in the SSL of melasma patients pre- and post-treatment is limited. For the first time, this study employed ultrahigh-performance liquid chromatography-triple quadrupole mass spectrometry (UHPLC-QTRAP MS) to explore the lipidomic changes in melasma patients before and after treatment that involved oral administration of tranexamic acid (TXA) and topical application of hydroquinone (HQ) cream.

## Methods

### Population

The Ethics Committee of Hangzhou Third People’s Hospital authorized the research protocol. The patients comprehensively understood the purpose of the experiment. The participants voluntarily participated and provided informed consent by signing a consent form. In total, 15 female melasma patients (aged 40.87 ± 1.45 years; range, 32–51 years) were recruited in this prospective study. Participants were selected the patients from Hangzhou Third People’s Hospital, and received melasma treatment at the Melasma Specialist Clinic between June and October 2022. Supplementary file 1 lists the criteria for inclusion and exclusion of participants in the study. Eligible patients were treated with a topical HQ cream (2% concentration, thinly applied to the melasma lesion area once in the morning and once in the evening) and oral TXA tablets (each tablet contained 0.5 g TXA, administered orally as half a tablet twice a day).

Each patient was followed up for 9 weeks, with data recorded during the first visit (T0), at 3-week intervals for the second visit (T1), and again at 9-week intervals for the third visit (T2). Skin lipid composition was measured twice for each patient (T0 and T1), and melasma severity and quality of life were assessed three times (T0, T1, and T2). The patients were asked throughout the study to use regulated medications and mild medical skincare products, prioritize moisturization, and practice sun protection. They were advised to avoid facial topical treatments, chemical peels, other lasers, and phototherapy.

### Evaluation of melasma severity and the impact on life

Two clinicians assessed the Melasma Area and Severity Index (MASI) scores T0, T1, and T2, each at or above the level of associate chief physicians and independent of the experimental designer and operator. In addition, the Melasma Quality of Life (MELASQoL) score was employed to assess the impact of melasma on the patient’s life. Furthermore, at T0 and T2, the melasma lesion area was imaged using reflection confocal microscopy (RCM) to observe alterations in epidermal melanin content, and dermoscopy was used to observe pigment and blood vessel distribution.

### Collection of lipid samples

Subjects were instructed to cleanse faces lightly with water and then wait for 30 min in a room maintained at a controlled temperature of 22–26℃ and humidity level of 40–60%. In the skin lesions of these patients, D-squame® test strips were used to collect epidermal stratum corneum samples. Individual strips were applied to each patient’s skin, pressed three times to ensure a tight fit, and removed at a constant speed after 3 min. Immediately after removing the strip from the patient’s skin, it was stored in the refrigerator at − 80℃ and subsequently analyzed for superficial lipids.

### The preparation of quality control (QC) samples

In each group, samples were combined to create a QC sample. These QC samples served to assess the instrument’s status prior to sample injection, to calibrate the system, and were periodically injected throughout the testing process to monitor the stability of the system over the course of the entire experiment. In this study, the instrument’s stability, the experiment’s repeatability, and the reliability of the data quality were systematically assessed across five different quality control measures (comparison of total ion chromatogram of QC samples, correlation map of QC samples, Hotelling’s T2 test for population samples, multivariate control chart of QC samples, and relative standard deviation of QC samples; the QC results are shown in Supplementary file 2).

### The preconditioning of samples

Following the gradual thawing of the samples at 4 °C, and 200 μL methanol, through vortex mixing. The appropriate samples were treated with the addition of 20 μL of an internal standard mixture and 800 μL of methyl tert-butyl ether (MTBE), which were thoroughly mixed using vortexing. The mixture was subjected to ultrasonic treatment in a water bath maintained at low temperature for 20 min, after which it was placed for 30 min at room temperature, then mixed with a 200 μL mass spectroscopic water vortex. The mixture was then centrifuged at 14,000 rpm for 15 min at a temperature of 4 °C. Next, the upper organic layer was carefully decanted and dried using nitrogen gas. During mass spectrometry, the mixture was re-dissolved with 200 μL 90% isopropyl alcohol/acetonitrile solution. After full swirled and centrifugation, the supernatant sample was analyzed.

### UHPLC-QTRAP MS analysis

On the UHPLC Nexera LC-30 A ultra-high performance liquid chromatography system (Shimadzu, Japan), the samples were separated using Kinetex C18 (Phenomenex; 2.6 μm; 2.1 mm × 100 mm) with HILIC NH2 columns (Phenomenex; 3 μm; 2.0 mm × 100 mm). The conditions of columns are showed in Supplementary file 3.

Throughout the analysis, the samples were kept in an automatic injector maintained at 10 °C. Continuous sample analysis was carried out in random order (to prevent errors arising from variations in the instrumental detection signals).

Electrospray Ionization (ESI) was used to analyzed the samples. The samples underwent separation via UHPLC, then AB 6500 + QTRAP mass spectrometer (AB SCIEX, MA, USA) was subsequently analyzed. The conditions for the ESI were as follows: Source temperature: 400℃. Ion Source Gas1: 50; Ion Source Gas2: 55; Curtain Gas: 35; Ion Spray Voltage: +5500 V or -4500 V (positive or negative modes, respectively), using MRM mode monitoring.

### Data processing and statistical analysis

Statistical analysis of the data was performed with SPSS version 26.0 (IBM Corp., Armonk, NY, USA) software, and GraphPad Prism 9.5 (GraphPad Software, Boston, MA, USA) was used to create the relevant figures. Measured data are presented as means ± standard deviations. A paired t-test was used for analysis when the data were normally distributed and showed homogeneity of variance. Otherwise, a paired rank test was used. Differences in MASI and MELASQoL scores at T0, T1, and T2 were compared and analyzed. *P* < 0.05 indicated statistical significance.

The MultiQuant OS software (SCIEX, Toronto, Canada) was used for peak extraction of the raw MRM data to obtain the ratio of the peak area between the sample and the internal standard. The content of each sample was then determined. Principal component analysis and supervised orthogonal partial least squares discriminant analysis (OPLS-DA) to determine the lipid composition at T0 and T1. The selection criteria involved variables with a variable influence on projection (VIP) value > 1 and a *P*-value < 0.05. VIP is a recognized metric that quantifies the significance of a variable (X) within an OPLS-DA model incorporating multiple components.

## Results

### MASI and MELASQoL scores before and after treatment

**Fig. 1:**
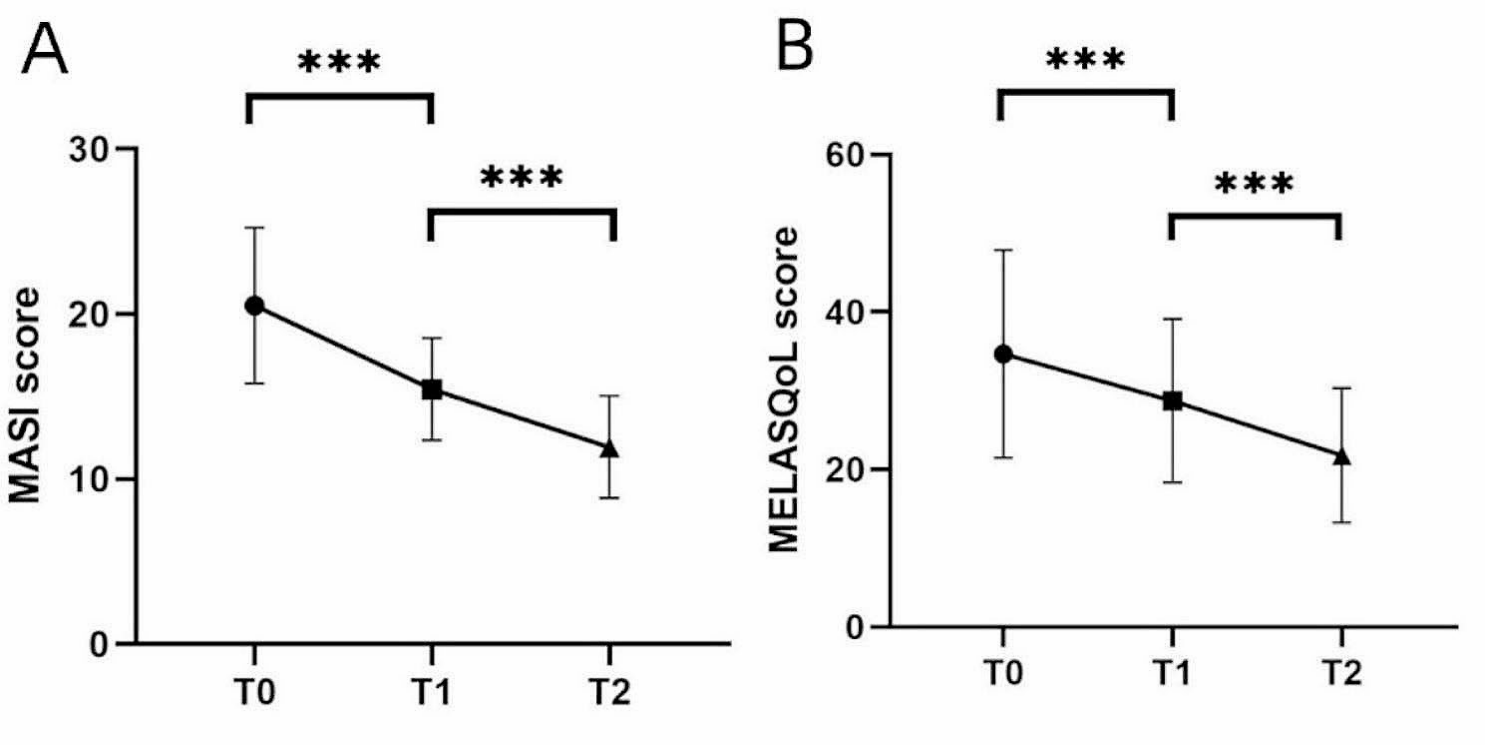
(**A**) Changes in Melasma Area and Severity Index (MASI) across different periods. (**B**) Changes in Melasma Quality of Life (MELASQoL) scores of patients across different periods. *** *P* < 0.001

Figure 1 illustrates the decrease in the MASI and MELASQoL scores observed before and after melasma treatment with oral TXA and topical HQ cream. The MASI and MELASQoL scores at T1 were markedly decreased compared to those at T0 (*P* < 0.001), and further reductions were observed at T2 compared to T1 (*P* < 0.001).

### RCM and dermoscopic images before and after melasma treatment

**Fig. 2:**
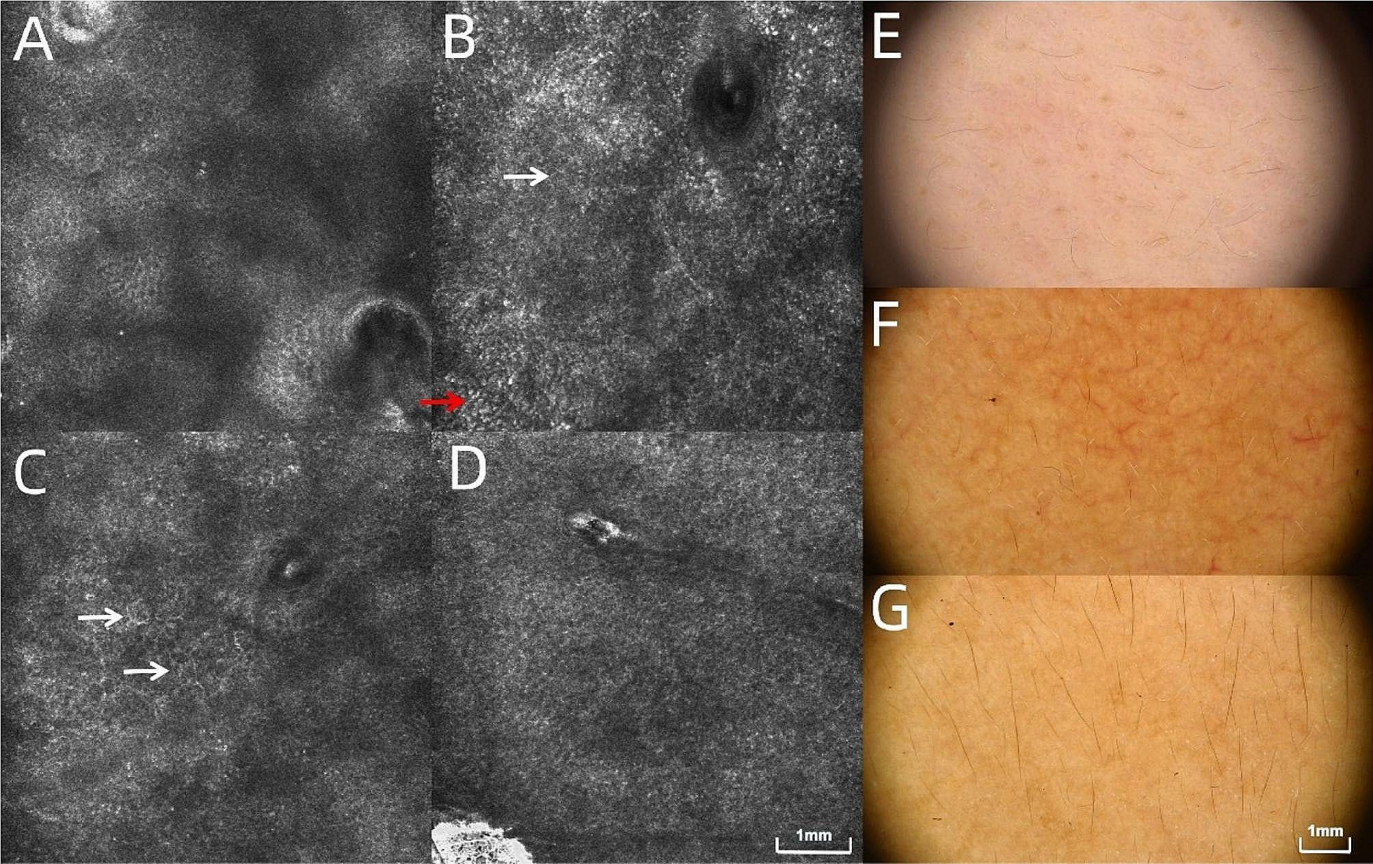
reflection confocal microscopy (RCM) and Dermoscopic images of normal facial skin and images before and after treatment of melasma with oral tranexamic acid (TXA) and topical hydroquinone (HQ) cream. (**A**) A normal facial RCM image (×30); (**B**) RCM image of melasma lesions at T0 (×30, the red arrows represent melanin particles and the white arrows represent melanin cells): the melanin content in the stratum corneum and basal layer of the lesions significantly increased compared to that of (**A**); (**C**) Dendritic cells in melasma lesions at T0 (×30, the white arrows represent dendritic cells); (**D**) RCM image of the melasma lesion area at T2 show that the melanin content was reduced compared to that at T0 (**B**), and no obvious dendritic cells are observed (×30); (**E**) Dermoscopic image of **a** normal face (×20); (**F**) Dermoscopic image of melasma lesions at T0 (×20): Uniform yellow–brown patches can be seen, with honeycomb or reticular distribution and linear or dendritic capillary networks; (**G**) Dermoscopic image of the melasma lesion area at T2 (×20): the color of the spots in the tan background is lighter than that in (**F**), and blood vessels are thinned and reduced in number (**F** and **G** originated from the same patients, **E** originated from healthy control)

The RCM images of melasma lesions at T0 indicated a significant increase (15/15; 100.0%) in melanin content in stratum corneum and basal layer. Dendritic cells in the lesions were present (9/15, 60.0%). At T2, melanin content decreased (11/15, 73.3%), and dendritic cells decreased or disappeared (6/9; 66.7%; Fig. 2A-D) compared to those at T0.

Compared with normal controls, dermoscopic images of melasma lesions at T0 showed uniform yellow-brown patches with a honeycomb or reticular distribution (15/15, 100.0%) and partially linear or dendritic capillary networks (11/15, 73.3%). By T2, the color of the spots on the yellow-brown background became lighter (13/15; 86.7%), and the blood vessels became thinner and reduced in number (7/11; 63.6%; Fig. 2E-G).

### Differential lipid analysis before and after oral TXA and topical HQ cream treatment

**Fig. 3:**
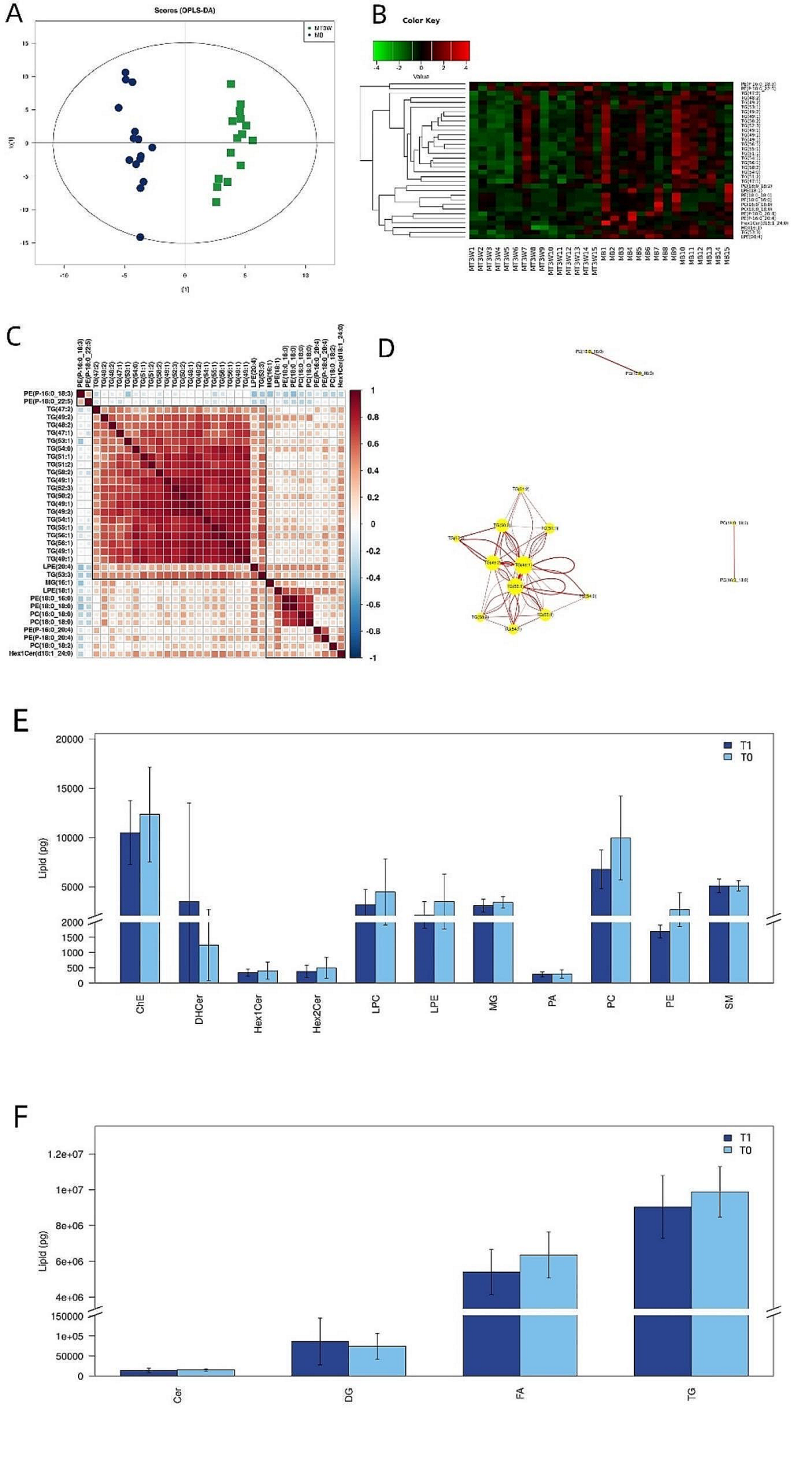
Multivariate data analysis of lipids before and after melasma treatment. (**A**) Scoring plot of OPLS-DA depicting the molecular variations between T0 and T1 lipids in melasma patients. (**B**) Heat map of 34 lipid species (*P* < 0.05; VIP > 1; Drawn in R language, visualized based on ggplot2). Differential color blocks placed at various locations signify the relative abundance of lipid molecules at those specific locations. A shade of red denotes higher expression levels, while a shade of green indicates lower expression levels. Lipid molecules displaying analogous expression profiles are grouped together in the same category on the left side. (**C**) Correlation matrix depicting the relationships between 34 lipid species (*P* < 0.05; VIP > 1; Drawn in R language, visualized based on ggplot2). The color scale ranges from red to blue, where red denotes a positive correlation and blue a negative one. The intensity of the color corresponds to the magnitude of the correlation coefficient’s absolute value. (**D**) Graphical representation of the network involving 34 lipid species (*P* < 0.05; VIP > 1; Visualized by Cytoscape Version 3.8.2). (**E**) and (**F**) represent the content changes of different lipid subclasses in lipidomics tests

The lipidomic analysis identified 15 lipid subclasses and 382 lipid molecules. By employing OPLS-DA, we were able to eliminate noise that was not pertinent to the classification information, thereby enhancing the analytical capabilities and overall efficacy of the model. Consequently, OPLS-DA analysis was carried out to pinpoint the crucial individual lipid species among the subclasses, and a score map was utilized to assess the variation in lipid composition between the two distinct sample sets. The separation between the two groups was well distinguished (Fig. 3A). The results revealed notable variations in epidermal SSL across various phases.

Applying multivariate data analysis, OPLS-DA elucidated the critical individual lipids that were most influential in distinguishing these subclasses; 34 lipid molecules exhibited significant differences between T1 and T0 (Fig. 3B; VIP > 1; *P* < 0.05). The specific quantity data are shown in Supplementary file 4, detected differences in lipid subclasses are shown in Fig. 3E-F. The fold change (FC) of the six lipid molecules was greater than 0.8 times (FC > 0.8; Table 1). The low content of some lipids had little influence on the VIP value; however, the disparity between T1 and T0 remained statistically significant for a subset of 12 lipids. (*P* < 0.05; FC > 0.7; Table 2).

**Table 1:** Lipid molecules with significant differences before and after melasma treatment (*P* < 0.05, VIP > 1; FC > 0.8)

Name	Class	Mass.info	Retention. Time (min)	column	FC		*P*-value	VIP
PE(P-16:0/18:3)	PE	696.5/277.2	7.15	Amino	1.267		< 0.01	2.208
PE(P-18:0/22:5)	PE	776.6/329.2	7.15	Amino	1.213		< 0.05	2.043
TG(49:2/FA18:2)	TG	834.8/537.5	8.82	C18	0.812		< 0.05	1.178
TG(58:2/FA18:1)	TG	960.9/661.6	10.54	C18	0.811		< 0.05	1.314
TG(47:1/FA14:0)	TG	808.7/563.5	8.64	C18	0.809		< 0.05	1.178
TG(54:0/FA16:0)	TG	908.8/635.5	10.44	C18	0.805		< 0.01	1.087

FC: fold change; VIP: variable influence on projection; PE: phosphatidylethanolamine; FA: fatty acid; TG: triglyceride

**Table 2:** Other significantly different lipid molecules before and after melasma treatment (*P* < 0.05; FC > 0.7)

Name	Class	Mass.info	Retention. Time (min)	column	FC	*P*-value	VIP
PE(18:1/18:1)	PE	742.5/281.2	7.15	Amino	0.887	< 0.05	0.956
TG(50:0/FA16:0)	TG	852.8/579.5	9.64	C18	0.880	< 0.05	0.875
TG(50:1/FA14:0)	TG	850.8/605.6	9.35	C18	0.825	< 0.05	0.980
TG(54:4/FA18:2)	TG	900.8/603.5	9.21	C18	0.817	< 0.01	0.959
PC(18:1/18:1)	PC	844.6/281.2	5.85	Amino	0.793	< 0.05	0.810
TG(52:0/FA20:0)	TG	880.8/551.5	10.07	C18	0.786	< 0.05	0.723
TG(53:3/FA17:0)	TG	888.8/601.5	9.32	C18	0.785	< 0.05	0.578
DHCer(d18:0/22:0)	DHCer	624.8/266.4	6.44	C18	0.752	< 0.05	0.869
LPC(20:3)	LPC	604.3/305.2	6.77	Amino	0.731	< 0.01	0.987
TG(52:4/FA22:4)	TG	872.8/523.5	8.80	C18	0.718	< 0.01	0.848
PE(P-18:0/20:1)	PE	756.6/309.3	7.15	Amino	0.711	< 0.05	0.664
ChE(20:4)	ChE	690.6/369.4	8.86	C18	0.705	< 0.05	0.581

FC: fold change; VIP: variable influence on projection; PE: phosphatidylethanolamine; TG: triglyceride; PC: phosphatidylcholine; DHCer: dihydroceramide; LPC: lysophosphatidylcholine; ChE: cholesteryl ester

The signaling pathway analysis of 34 lipid molecules with significant differences in melasma lesions showed a positive correlation (Fig. 3C), including triglycerides (TGs) such as TG (49:1), TG (49:2), and TG (50:2). Within these, TG (56:1) was found to play a significant role in the observed process (Fig. 3D).

**Fig. 4:**
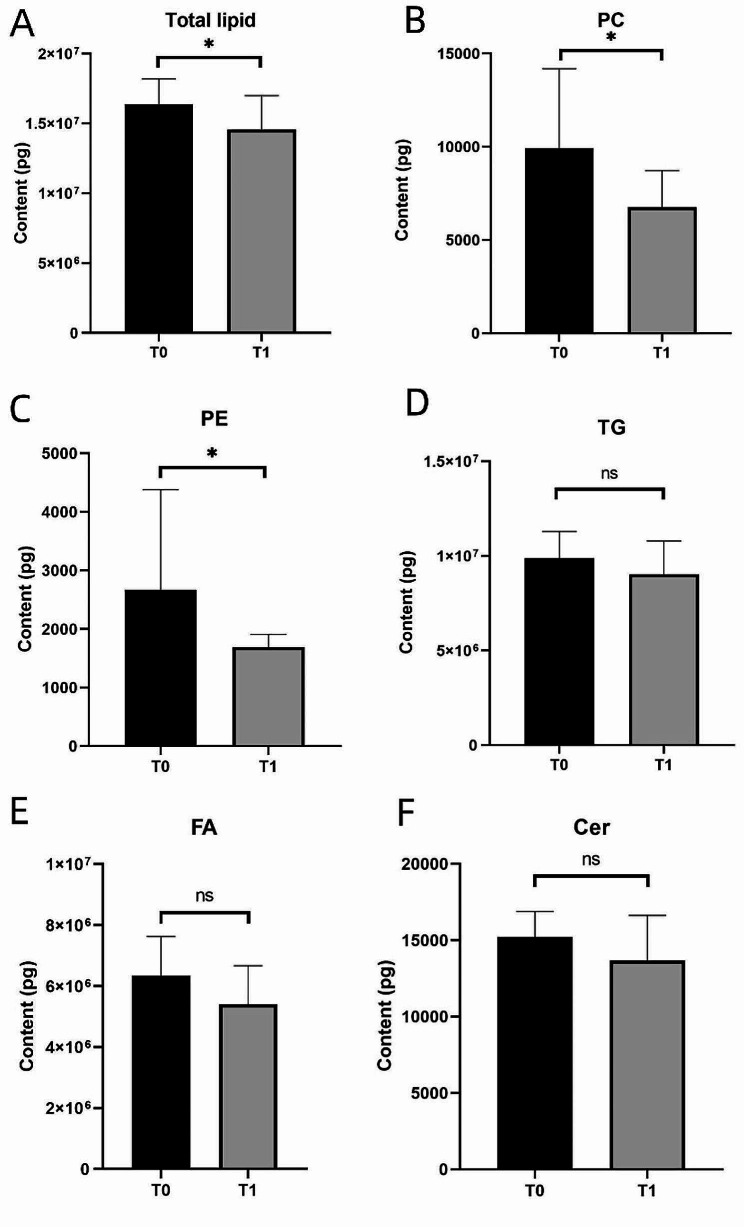
Comparison of the expression profiles of total lipids and certain lipid classes prior to and following melasma treatment. (**A**), (**B**), (**C**), (**D**), (**E**), and (**F**) show the comparison of total lipids, phosphatidylcholine (PC), phosphatidylethanolamine (PE), triglyceride (TG), fatty acid (FA), and ceramide (CER) content before and after treatment, respectively. After 3 weeks of treatment with oral TXA and topical HQ cream, the expression of total lipids, PC, and PE in the skin was significantly reduced (*P < 0.05), and the expression of TG, FA, and CER decreased, but not significantly (ns *P* > 0.05)

Expressions of total lipids, phosphatidylcholine (PC), and phosphatidylethanolamine (PE) significantly decreased at T1 compared with T0 (*P* < 0.05), while the expression of TGs, fatty acids (FAs), and CERs decreased, although these variations failed to achieve statistical significance (*P* > 0.05; Fig. 4).

## Discussion

Treatment approaches for melasma include topical or oral drugs, lasers, chemical exfoliation, and camouflaged cosmetics. The participants received oral TXA (250 mg twice daily) in conjunction with topical HQ cream (applied twice daily) as the treatment regimen for melasma. After 3 weeks of treatment, the mean MASI score decreased from the baseline value of 18.6 (17.0–23.4) to 14.6 (12.4–17.2), and after 9 weeks of treatment, the MASI score further decreased to 11.0 (9.6–14.4; *P* < 0.001). The side effects of the treatment, including slight erythema and tingling from the HQ cream, were tolerated with medication use. Studies have demonstrated that this treatment method is safe and effective and that its efficacy increases with treatment duration. Shihab et al. confirmed the effectiveness of this treatment, reporting a decrease in the modified MASI score from 8.96 ± 2.45 to 4.00 ± 1.60 3 months after treatment [13].

The mean MELASQoL scores decreased from the baseline (34.67 ± 13.19) to 28.73 ± 10.38 after 3 weeks and further to 21.80 ± 8.54 after 9 weeks (*P* < 0.001). The findings revealed a correlation between the decrease in skin lesions and the enhancement of the life quality.

The SSL is crucial for the skin’s barrier function. Based on their source, lipids are divided into sebaceous glands and intercellular lipids. The former includes TGs, FAs, and squalene, whereas the latter includes CERs, cholesterol, and free FAs. Both types combine with sweat to form sebum membranes. It is crucial for preserving skin health, protecting against external aggressors, and controlling the metabolic activity of skin cells while maintaining the equilibrium of the skin microbiota [14]. Alterations in the lipid profile of the stratum corneum may undermine the skin’s barrier integrity, leading to an increase in transepidermal water loss [5, 15]. Chung et al. reported the downregulation of lipid genes related to the PPAR signaling pathway and the upregulation of genes related to cuticle barrier function [16] in melasma lesions, revealing significant differences from those in normal skin. Xu et al. explored the differences in the content and types of lipids between melasma-affected areas and unaffected areas. The researchers discovered that the levels of total lipids, phosphatidic acid, and CER in the melasma-affected areas are markedly higher than those in normal skin [2].

In this study, the total lipid level and PC and PE content in melasma lesions decreased after treatment (*P* < 0.05). PC and PE participate in cellular signaling and are fundamental components of cell membranes, maintaining their structure and stability. Recent research revealed that PC and PE can lower lipofuscin levels in cells within the stratum corneum, with PC playing a more significant role [17]. Therefore, owing to the increase in pigment content in melasma lesions, the compensation of the two can be increased to meet the body’s need to reduce more pigment. Phospholipid compounds are a class of low-toxicity, non-irritating osmotic agents [14]. Their content is higher in melasma lesion areas, making the skin more permeable, and their ability to resist external stimulation may decline. As a result of melasma-induced apoptosis, PE can further induce apoptosis [18]. Before treatment, melasma lesions had a high PE content, stemming from excessive apoptosis, which could lead to further apoptosis of the skin epidermal cells, resulting in compromised skin barrier function. After treatment, the expression of PC and PE in the skin decreased, indicating an improvement in skin barrier function.

TG is an important lipid component secreted by the laminae that maintains skin barrier function. It suppresses the production of matrix metalloproteinase-1, cyclooxygenase-2, and interleukin-1β to protect HaCaT cells after ultraviolet irradiation. Thus, TG is crucial for healing skin injury following ultraviolet radiation exposure [14]. This study revealed that the total TG content in melasma lesions decreased after 3 weeks of treatment (*P* > 0.05), and the content of nine TG molecules decreased significantly (*P* > 0.05, FC > 0.7), with four types having VIP content > 1. This indicated that skin inflammation at the melasma lesion decreased after treatment. A comparative study of SSL discrepancies between infants with AD and healthy infants highlighted a considerable increase in triglyceride content within the lipid profiles of infants with AD [14]. CER is composed of sphingosine and long-chain fatty acids, which are the primary lipid constituents of epidermal cells. A reduction in both these factors leads to transdermal water loss and increased barrier dysfunction [19]. In this study, the FA and CER contents decreased after treatment (*P* > 0.05), which was presumed to be related to the topical HQ cream.

### Strengths and limitations

As of now, this study represents the initial analysis of alterations in the SSL before and after melasma treatment using oral TXA and topical HQ cream. During this investigation, SSL levels in melasma-affected skin were compared, both before and after treatment, and the influence of variable factors was reduced as much as possible. One of the limitations of this study was that patients with melasma were not grouped, and the role of lipid components in different severities of melasma could not be determined. Additionally, the limited sample size meant that the observed changes in the expression of specific lipids did not reach statistical significance.

## Conclusions

This study revealed alterations in SSL composition after effective treatment of melasma, suggesting a compensatory role for lipids in melasma barrier function. More attention should be paid to the balance between lipid barriers in the daily care of patients with melasma. Analysis of the 34 differentially expressed lipids indicated that PC and PE were potential differentially expressed lipids. Further research is required to explore the mechanism involving SSL and the lipid barrier that affects the occurrence of melasma.

### Electronic supplementary material

Below is the link to the electronic supplementary material.

Supplementary Material 1

Supplementary Material 2

Supplementary Material 3

Supplementary Material 4

## Data Availability

No datasets were generated or analysed during the current study.

## Abbreviations

AD: Atopic dermatitis
CER: Ceramide
ChE: Cholesteryl ester
DHCer: Dihydroceramide
ESI: Electrospray Ionization
FC: Fold change
HQ: Hydroquine
LC-MS/MS: Liquid chromatography-mass spectrometry
LPC: Lysophosphatidylcholine
MASI: Melasma Area and Severity Index
MELASQoL: Melasma Quality of Life
OPLS-DA: Orthogonal partial least squares discriminant analysis
PC: Phosphatidylcholine
PE: Phosphatidylethanolamine
QC: Quality control
RCM: Reflection confocal microscopy
SSL: Skin surface lipids
TG: Triglyceride
TXA: Tranexamic acid
UHPLC-QTRAP MS: Ultrahigh-performance liquid chromatography-triple quadrupole mass spectrometry
VIP: Variable influence on projection
