# Supplementary material for: Comparative study of melasma in patients before and after treatment based on lipomics
Source: Lipids Health Dis. 2024 May 11;23:138. doi: 10.1186/s12944-024-02130-z (PMC11088129; doi:10.1186/s12944-024-02130-z)
Supplement: Supplementary file 1 — Supplementary Material 1 [file 12944_2024_2130_MOESM1_ESM.docx]

Supplementary file 1: The inclusion and exclusion criteria for the study population.

Inclusion criteria:

(1) Untreated patients with melasma over 18 years of age;

(2) The Melasma Area and Severity Index (MASI) score is greater than 8 points.

Exclusion criteria:

(1) Pregnant and lactating women;

(2) Patients who have given birth or received hormone therapy in the past 12 months;

(3) Continuous use of oral contraceptives, endocrine affecting drugs, phototoxic drugs (such as minocycline, methotrexate, phenytoin sodium, etc.);

(4) Patients with a history of thromboembolism or abnormal bleeding;

(5) Patients with other related endocrine diseases or pigmentation diseases;

(6) Patients with skin infections or skin tumors;

(7) Patients known to be allergic to tranexamic acid (TXA) or hydroquinone (HQ) cream;

(8) Patients with a history of excessive sun exposure within 1 month;

(9) Patients with mental illness who cannot cooperate, fail to continue follow-up or have low compliance.
