# Supplementary material for: Comparative study of melasma in patients before and after treatment based on lipomics
Source: Lipids Health Dis. 2024 May 11;23:138. doi: 10.1186/s12944-024-02130-z (PMC11088129; doi:10.1186/s12944-024-02130-z)
Supplement: Supplementary file 2 — Supplementary Material 2 [file 12944_2024_2130_MOESM2_ESM.docx]

Supplementary file 2: Quality control (QC) results


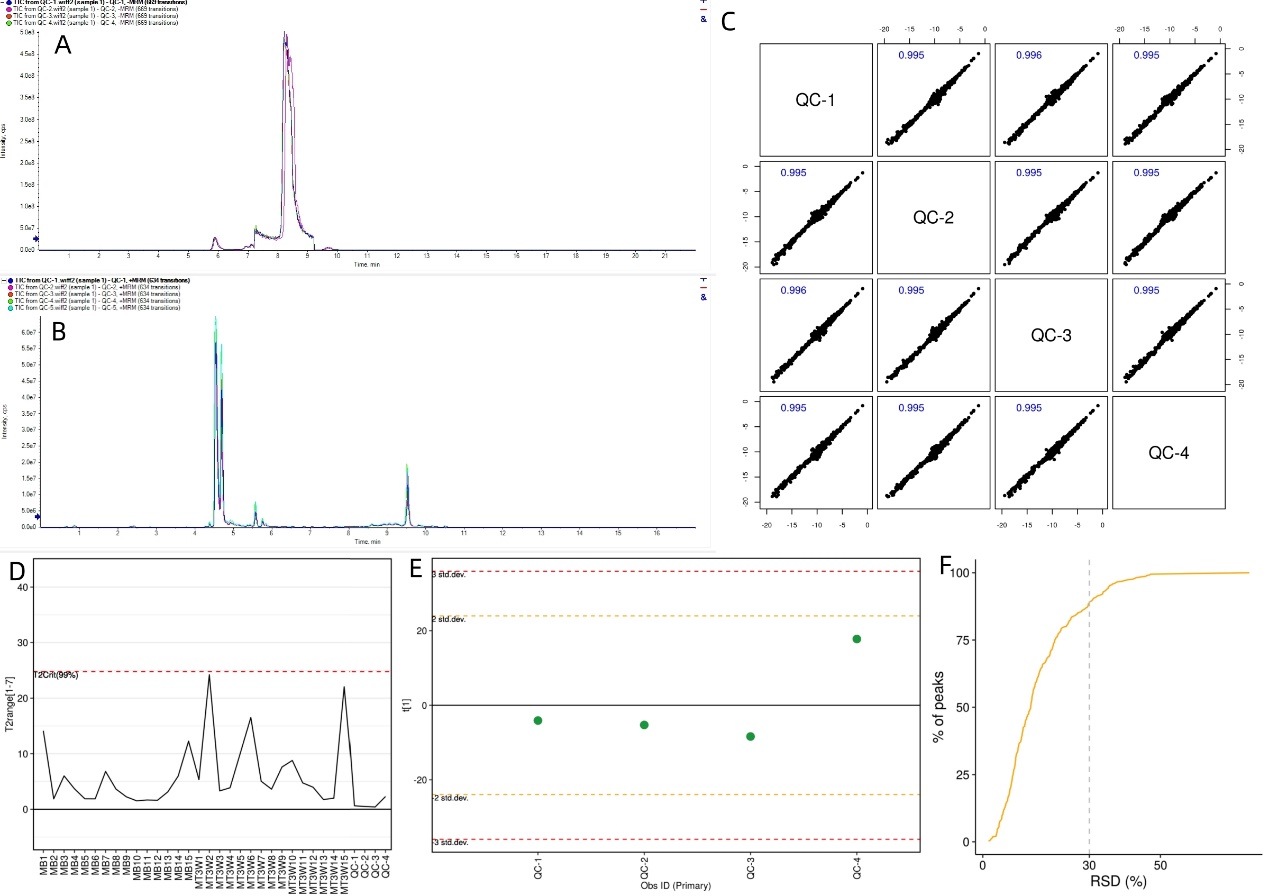


(A) and (B): Comparison of total ion chromatogram (C18 column and Amino column, respectively) of QC samples. Results showed that the response intensity and retention time of each chromatographic peak basically overlap, indicating that the variation caused by instrument error is small in the whole experiment process. (C): Correlation map of QC samples. Pearson correlation analysis was performed on QC samples. Generally, correlation coefficient greater than 0.9 indicates good correlation. The results showed that the correlation coefficients between QC samples are above 0.9, indicating that the experimental repeatability is good. (D): Hotelling's T2 test for population sample. Hotelling's T2 test tests the sample through multivariate modeling, defining 95% or 99% confidence intervals, which can be used for the diagnosis of outlier samples. Results showed that all QC samples were within 99% confidence interval, indicating good repeatability of the experiment. (E): Multivariate control chart of QC sample. Each point in the multivariable control chart represents a QC sample, and the X-axis is the loading order of all QC samples. The points in the diagram fluctuate up and down due to the fluctuating state of the instrument. Generally, plus or minus 3 standard deviations is the normal range. The experimental results showed that the fluctuation of QC samples is within the range of plus or minus 3 standard deviations, reflecting that the fluctuation of the instrument is within the normal range, and the data can be used for subsequent analysis. (F): Relative standard deviation (RSD) of QC sample. The smaller the RSD of ion peak abundance of QC sample, the better the stability of the instrument, which is an important index to reflect the quality of data. In this experiment, the proportion of the peak number of QC samples with RSD≤30% accounted for more than 80% of the total peak number of QC samples, indicating that the instrument analysis system has good stability, and the data can be used for subsequent analysis.
