# Supplementary material for: Comparative study of melasma in patients before and after treatment based on lipomics
Source: Lipids Health Dis. 2024 May 11;23:138. doi: 10.1186/s12944-024-02130-z (PMC11088129; doi:10.1186/s12944-024-02130-z)
Supplement: Supplementary file 3 — Supplementary Material 3 [file 12944_2024_2130_MOESM3_ESM.docx]

Supplementary file 3: The conditions of C18 column and HILIC column.

C18 column: Column temperature 45℃; Flow rate 0.35 mL/min; Mobile phase composition A: 70% acetonitrile + 30% water + 5 mM ammonium acetate, B: isopropyl alcohol solution; Gradient elution procedure: 0–5 min, B linear change from 20% to 60%, 5–13 min, B linear change from 60% to 100%, 13–17 min, B maintained at 20%.

HILIC column: Column temperature 40℃; Flow rate 0.4 mL/min; Mobile phase composition A: 2 mM ammonium acetate + 50% methanol + 50% acetonitrile, B: 2 mM ammonium acetate + 50% acetonitrile + 50% water, 0–3 min, B maintained at 3%, 3–13 min, B linearly changed from 3% to 100%, 13–17 min, B maintained at 100%, 17–22 min, B maintained at 3%.
