# Supplementary material for: Comparative study of melasma in patients before and after treatment based on lipomics
Source: Lipids Health Dis. 2024 May 11;23:138. doi: 10.1186/s12944-024-02130-z (PMC11088129; doi:10.1186/s12944-024-02130-z)
Supplement: Supplementary file 4 — Supplementary Material 4 [file 12944_2024_2130_MOESM4_ESM.docx]

Supplementary file 4: The specific quantity data of lipid subclasses.

| class | Cer | ChE | DG | DHCer | FA | Hex1Cer | Hex2Cer | LPC | LPE | MG | PA | PC | PE | SM | TG |
| --- | --- | --- | --- | --- | --- | --- | --- | --- | --- | --- | --- | --- | --- | --- | --- |
| Pvalue | 0.33122 | 0.228431 | 0.483456 | 0.390089 | 0.052624 | 0.439181 | 0.290022 | 0.192098 | 0.100031 | 0.185814 | 0.720195 | 0.013693 | 0.037161 | 0.987598 | 0.155145 |
| Ratio | 0.897327 | 0.851266 | 1.167149 | 2.825891 | 0.851539 | 0.845722 | 0.773656 | 0.717841 | 0.614253 | 0.910151 | 0.949905 | 0.679651 | 0.633948 | 1.000694 | 0.914085 |
| T1A | 9819.018 | 11817.05 | 42894.52 | 611.8577 | 5403640 | 458.9736 | 392.1039 | 4458.011 | 1534.547 | 2767.115 | 284.0564 | 5043.875 | 1431.486 | 5732.52 | 7321078 |
| T1B | 9811.83 | 20342.69 | 41732.03 | 576.7961 | 3414094 | 348.1796 | 226.4436 | 4785.197 | 2622.173 | 2480.569 | 536.1195 | 11940.63 | 1953.126 | 5658.635 | 9204153 |
| T1C | 10903.38 | 8372.178 | 88629.32 | 655.0799 | 5425196 | 273.8874 | 892.0571 | 2792.505 | 2553.321 | 3822.062 | 212.7259 | 6623.005 | 1992.672 | 5175.318 | 7142048 |
| T1D | 11522.06 | 8100.114 | 85598.79 | 625.1595 | 5307521 | 256.9856 | 429.7783 | 3197.412 | 1526.99 | 2979.128 | 284.0564 | 6558.499 | 1485.73 | 5235.43 | 7524416 |
| T1E | 22307.5 | 7512.124 | 98324.71 | 923.7461 | 5427007 | 390.8242 | 549.0775 | 2149.128 | 1575.761 | 3313.298 | 268.4525 | 6324.609 | 1565.074 | 5137.189 | 7066820 |
| T1F | 19242.88 | 7410.167 | 223077.3 | 848.6683 | 5412083 | 462.3791 | 378.5203 | 3412.762 | 1860.253 | 3645.222 | 284.0564 | 9705.744 | 2031.301 | 6350.665 | 10683306 |
| T1G | 11303.22 | 10917.56 | 68323.88 | 557.5817 | 5804989 | 287.7967 | 224.2335 | 7427.718 | 6734.886 | 3571.891 | 179.7341 | 6412.818 | 2011.203 | 4445.508 | 13703804 |
| T1H | 6367.155 | 10908.56 | 46969.38 | 487.7961 | 5403640 | 175.9906 | 88.72563 | 2049.383 | 1297.007 | 1872.403 | 284.0564 | 4701.66 | 1455.043 | 4348.84 | 9514326 |
| T1I | 5834.877 | 8514.611 | 47767.14 | 427.373 | 3794194 | 157.7655 | 74.76445 | 1637.871 | 1291.207 | 1972.172 | 284.0564 | 4631.112 | 1494.283 | 3728.431 | 7622950 |
| T1J | 14829.54 | 9793.55 | 66736.56 | 39536.6 | 5403640 | 293.3864 | 331.7051 | 1687.691 | 1375.482 | 2982.968 | 284.0564 | 5824.813 | 1531.849 | 5204.448 | 9435622 |
| T1K | 22484.51 | 10186.55 | 41910.85 | 1316.284 | 4749588 | 416.0327 | 378.6517 | 2982.787 | 2219.089 | 3447.05 | 232.0623 | 7710.035 | 1779.976 | 4594.578 | 8223439 |
| T1L | 18215.99 | 9600.151 | 79891.26 | 921.7087 | 5403640 | 541.0653 | 629.7993 | 3694.112 | 2325.31 | 2898.635 | 366.4295 | 8052.676 | 1749.414 | 6122.701 | 8384952 |
| T1M | 17081.4 | 12570.86 | 81030.22 | 991.4654 | 5260778 | 516.1591 | 382.6533 | 2602.219 | 1708.91 | 2959.405 | 177.9375 | 6741.749 | 1584.503 | 4866.306 | 9529033 |
| T1N | 9483.832 | 12580.76 | 57599.68 | 3296.881 | 5543351 | 250.4429 | 323.0026 | 2034.278 | 1466.418 | 3852.286 | 284.0564 | 5754.312 | 1710.205 | 4837.945 | 10364384 |
| T1O | 15737.19 | 8922.977 | 225025 | 1051.018 | 9301236 | 291.603 | 412.0354 | 3253.058 | 2211.572 | 3992.966 | 298.9898 | 5315.693 | 1589.45 | 5102.884 | 9758377 |
| T0A | 20379.05 | 9405.202 | 33799.07 | 1160.776 | 4791630 | 1375.743 | 515.7072 | 4646.668 | 2901.612 | 2701.352 | 299.0365 | 13407.12 | 3223.497 | 5324.6 | 10900005 |
| T0B | 13041.59 | 8746.484 | 48675.44 | 832.9547 | 5050900 | 396.0058 | 790.0573 | 2612.744 | 2250.605 | 2989.021 | 299.0365 | 6814.059 | 1707.898 | 5239.244 | 8259719 |
| T0C | 15238.7 | 13970.21 | 147414.2 | 970.1051 | 9068693 | 503.5197 | 1470.609 | 5559.248 | 4473.541 | 4841.087 | 508.6289 | 6895.633 | 1892.442 | 5824.647 | 9765687 |
| T0D | 15940.14 | 10570.29 | 44562.64 | 983.3518 | 7296721 | 511.7446 | 357.4951 | 4282.99 | 3514.967 | 3538.839 | 206.0754 | 9370.581 | 3036.549 | 6337.526 | 9344466 |
| T0E | 20103.46 | 9983.227 | 73402.83 | 1076.794 | 5937941 | 291.3209 | 540.8013 | 2923.408 | 2091.179 | 2920.792 | 655.5845 | 11059.75 | 1836.721 | 5042.437 | 10225675 |
| T0F | 14964.96 | 13158.5 | 64042.83 | 840.2883 | 7260995 | 320.4464 | 921.497 | 3390.707 | 2393.043 | 3150.501 | 217.9995 | 6670.931 | 2038.734 | 4838.91 | 9279881 |
| T0G | 13243.08 | 10549.82 | 41792.6 | 787.2586 | 4906680 | 307.6608 | 345.6124 | 15670.1 | 11466.6 | 2738.611 | 157.8081 | 13428.25 | 3037.504 | 5133.676 | 8118226 |
| T0H | 16493.94 | 11163.63 | 41293.14 | 643.0799 | 5211114 | 200.2936 | 154.5154 | 2672.031 | 2263.672 | 3912.201 | 188.4045 | 5269.81 | 1738.326 | 4654.829 | 8259169 |
| T0I | 16977.96 | 28455.45 | 88824.78 | 898.6482 | 5267567 | 349.418 | 176.6628 | 3844.195 | 4302.921 | 3102.767 | 178.6374 | 12968.41 | 3693.194 | 5687.395 | 12866735 |
| T0J | 11468.75 | 10593.53 | 77801.34 | 6537.692 | 6708494 | 332.7357 | 175.8681 | 5395.702 | 1911.017 | 3304.686 | 317.2264 | 12224.72 | 2009.603 | 4897.698 | 11514817 |
| T0K | 11764.39 | 14848.34 | 61243.58 | 630.0054 | 6013189 | 355.367 | 322.0528 | 2492.408 | 1958.121 | 3549.13 | 299.0365 | 7966.991 | 2164.861 | 5217.932 | 11401827 |
| T0L | 13308.55 | 9553.888 | 89579.08 | 703.6562 | 7597053 | 216.1006 | 446.4293 | 2516.033 | 1515.585 | 3216.918 | 407.1828 | 7232.198 | 1534.833 | 4443.928 | 9429100 |
| T0M | 18937.6 | 10051.13 | 64371 | 1083.145 | 5379282 | 370.1366 | 347.4482 | 2256.25 | 1679.461 | 3119.721 | 183.6733 | 8263.532 | 1928.807 | 4493.428 | 9950977 |
| T0N | 15298.42 | 12981.82 | 121223 | 877.7259 | 6878993 | 217.8116 | 379.0774 | 2824.428 | 1817.379 | 3980.877 | 268.3266 | 6070.067 | 1778.555 | 4425.78 | 10675133 |
| T0O | 11233.67 | 11045.62 | 111953 | 668.8062 | 7816729 | 307.4313 | 441.3021 | 6008.872 | 8049.229 | 4086.757 | 298.8904 | 21465.73 | 8390.147 | 4926.29 | 8220904 |
| Pvalue | 0.33122 | 0.228431 | 0.483456 | 0.390089 | 0.052624 | 0.439181 | 0.290022 | 0.192098 | 0.100031 | 0.185814 | 0.720195 | 0.013693 | 0.037161 | 0.987598 | 0.155145 |
| Ratio | 0.897327 | 0.851266 | 1.167149 | 2.825891 | 0.851539 | 0.845722 | 0.773656 | 0.717841 | 0.614253 | 0.910151 | 0.949905 | 0.679651 | 0.633948 | 1.000694 | 0.914085 |
| Mean_T1 | 13662.96 | 10503.33 | 86367.37 | 3521.868 | 5403640 | 341.4314 | 380.9034 | 3210.942 | 2153.528 | 3103.811 | 284.0564 | 6756.082 | 1691.021 | 5102.76 | 9031914 |
| Mean_T0 | 15226.28 | 12338.48 | 73998.57 | 1246.286 | 6345732 | 403.7157 | 492.3424 | 4473.052 | 3505.929 | 3410.217 | 299.0365 | 9940.519 | 2667.445 | 5099.221 | 9880821 |
| Sd_T1 | 5352.634 | 3209.024 | 58903.02 | 9987.352 | 1264317 | 118.2076 | 204.9865 | 1496.294 | 1345.718 | 650.96 | 84.82058 | 1966.606 | 216.5385 | 687.1772 | 1757015 |
| Sd_T0 | 2973.681 | 4797.82 | 32866.65 | 1473.133 | 1285235 | 283.7484 | 343.708 | 3336.89 | 2769.706 | 584.5592 | 136.0733 | 4255.665 | 1714.595 | 539.8529 | 1406071 |
